# *Astyanax* surface and cave fish morphs

**DOI:** 10.1186/s13227-020-00159-6

**Published:** 2020-07-11

**Authors:** William R. Jeffery

**Affiliations:** Department of Biology, University of Maryland, College Park, MD 20742 USA

**Keywords:** *Astyanax mexicanus*, Surface fish, Cavefish, Genetic approach, Development, Behavior, Regeneration, Metabolic processes, Biomedicine, Evolution

## Abstract

The small teleost fish *Astyanax mexicanus* has emerged as an outstanding model for studying many biological topics in the context of evolution. A major attribute is conspecific surface dwelling (surface fish) and blind cave dwelling (cavefish) morphs that can be raised in the laboratory and spawn large numbers of transparent and synchronously developing embryos. More than 30 cavefish populations have been discovered, mostly in northeastern Mexico, and some are thought to have evolved independently from surface fish ancestors, providing excellent models of parallel and convergent evolution. Cavefish have evolved eye and pigmentation regression, as well as modifications in brain morphology, behaviors, heart regenerative capacity, metabolic processes, and craniofacial organization. Thus, the *Astyanax* model provides researchers with natural “mutants” to study life in the challenging cave environment. The application of powerful genetic approaches based on hybridization between the two morphs and between the different cavefish populations are key advantages for deciphering the developmental and genetic mechanisms regulating trait evolution. QTL analysis has revealed the genetic architectures of gained and lost traits. In addition, some cavefish traits resemble human diseases, offering novel models for biomedical research. *Astyanax* research is supported by genome assemblies, transcriptomes, tissue and organ transplantation, gene manipulation and editing, and stable transgenesis, and benefits from a welcoming and interactive research community that conducts integrated community projects and sponsors the International Astyanax Meeting (AIM).

## Natural habitat and life cycle

**Fig. 1:**
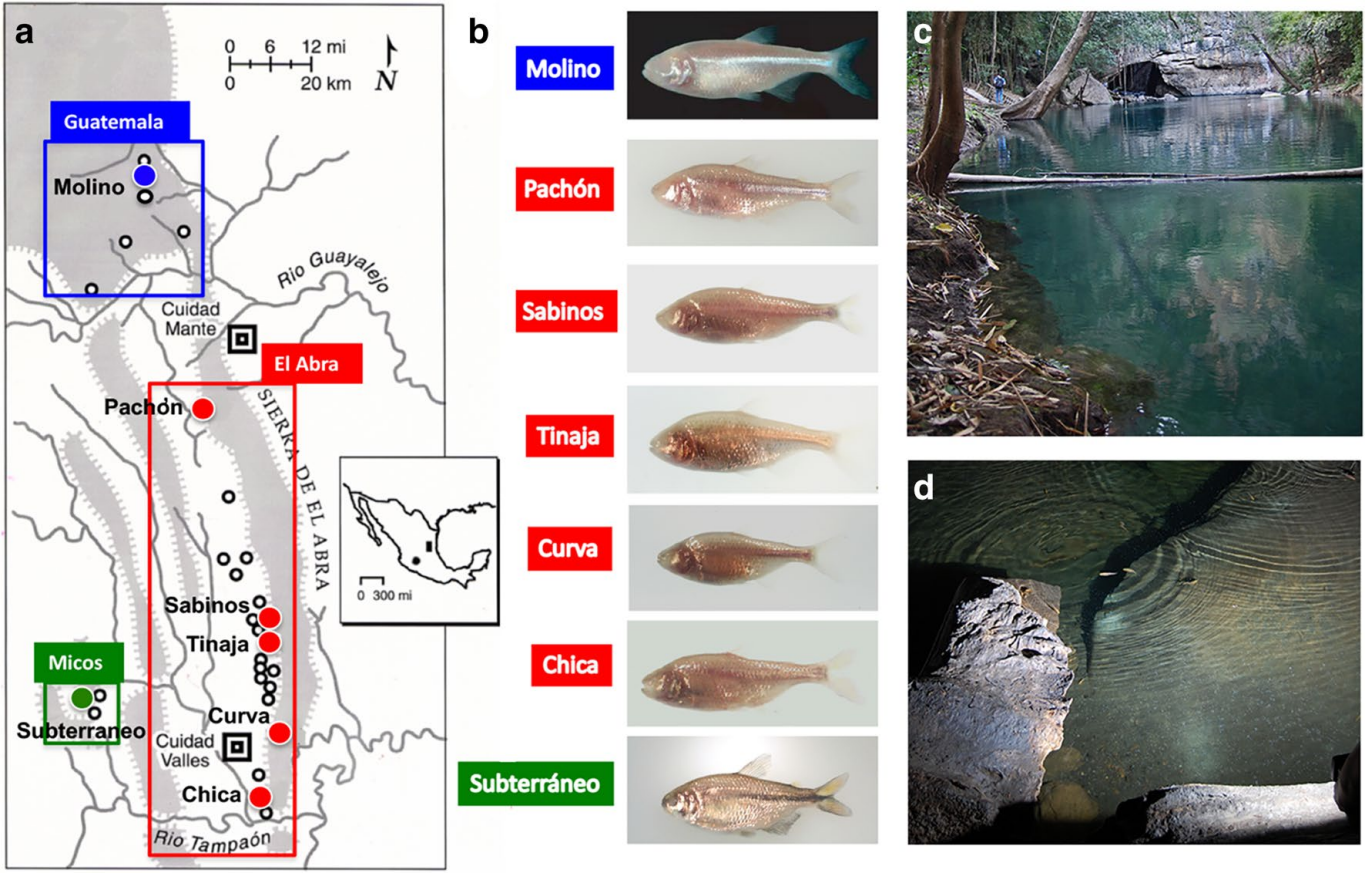
Astyanax caves, cavefish populations, and habitats. **a** A map showing the distribution of caves in the El Abra region of Tamaulipas and San Luis Potosí, Mexico. Boxes outlined in blue, red, and green show locations of Astyanax caves (black outlined and colored spheres) in the Sierra de Guatemala, Sierra de El Abra, and Micos regions, respectively. Inset: Mexico map showing the locations of *A. mexicanus* cavefish in the El Abra epicenter (right shaded rectangle) and *A. aeneus* cavefish in Guerrero (left shaded sphere). **b** Most frequently studied cavefish populations in the Guatemala (blue label), El Abra (red labels), and Micos (green label) regions. **c** A surface fish habitat at El Nacimiento del Río Choy. **d** A cavefish habitat in El Sótano de Las Piedras

*Astyanax mexicanus* is a small freshwater fish with a surface-dwelling morph (surface fish) and multiple cave-dwelling morphs (cavefish). Surface fish range widely in streams of southern Texas and northeastern Mexico (Fig. 1c). *A. mexicanus* is closely related to *A. aeneus*, which is distributed further south into Central America. The *A. mexicanus* cave morphs are centered in the Sierra de El Abra in Tamaulipas and San Luis Potosí, Mexico (Fig. 1a). Here cavefish have been recorded living in pools in about 30 caves (Fig. 1d) [1, 2]. The most studied cavefish are from Pachón and Tinaja caves in the El Abra region and Molino cave in the adjoining Sierra de la Guatemala (Fig. 1b). Two cavefish populations related to *A. aeneus* are also present in Guerrero, Mexico. *A. mexicanus* surface fish and cavefish diverged during the past 200,000 years [3, 4]. The cavefish populations have complex evolutionary histories impacted by introgression and parallel or convergent evolution [5].

Fertilization and development are external in *A. mexicanus*, and adults produce hundreds of eggs in a single spawn. The morphs have yolky eggs, meroblastic discoidal cleavage, and exhibit embryonic development resembling zebrafish [6]. Embryogenesis is rapid, gastrulation begins at about 6 hpf, and hatching at about 1 dpf (23 °C). Fry undergo metamorphosis in about a month, and adults reach sexual maturity in 6–8 months. Adults grow to 8–10 mm in length and have a lifespan of 10 or more years.

## Field collection and laboratory culture

Surface fish are collected by cast and seine nets or live traps. Experience in cave exploration is compulsory for cavefish collection. Most caves harboring cavefish populations are entered through deep pits, requiring specialized equipment, although Pachón and Tinaja caves have horizontal entrances, permitting easier access. Cavefish can be collected with hand-held nets or live traps. Some cavefish populations are readily sampled because they exhibit vibration attraction behavior (VAB), and thus swim toward the vibrations caused by nets dipped into the water [7]. Most researchers do not rely on natural collections. The morphs can usually be obtained by contacting an *Astyanax* research laboratory. Cavefish are sometimes available for purchase in pet stores, but these animals are not recommended for research. They are derived from Chica cave, which experiences invasion of surface fish during seasonal floods [1, 2] and therefore have a mosaic genetic background.

The morphs are cultured separately at 23–25 °C under a 14–10 h light–dark photoperiod [8, 9]. They are fed daily with tetra flakes supplemented by living invertebrates (Additional file 1: Movie S1 and Additional file 2: Movie S2). Aquarium setups are used with constantly flowing pure water, similar to those for raising zebrafish, although tank sizes are generally larger (40 L). Spawning of healthy fish can be induced by increasing the water temperature and the frequency of feeding. Surface fish begin to spawn a few hours after “lights off”, and cavefish, which respond to light despite blindness, delay spawning until the middle of the dark period. The morphs can be cultured under asynchronized light–dark periods to allow spawning together. Healthy adults raised in the laboratory generally spawn throughout the year for 3–5 years. Fry are raised in large numbers in smaller tanks and fed brine shrimp or rotifers.

## Major interests and research questions

**Fig. 2:**
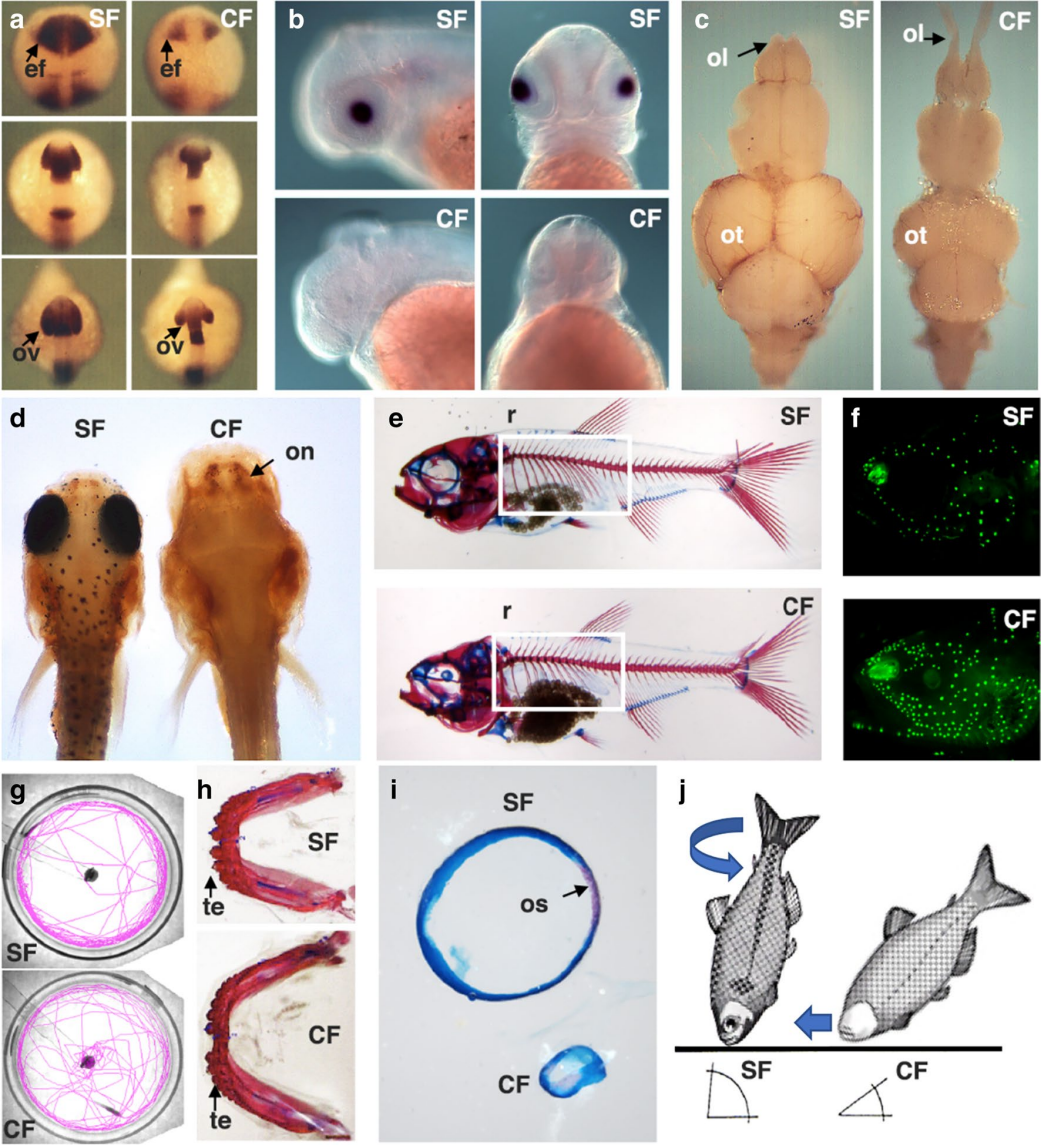
Examples of trait differences between surface fish (SF) and cavefish (CF). **a** In situ hybridization showing differences in *pax6* expression from the neural plate (top) to the late optic vesicle stages (below). *ef* eye field, *ov* optic vesicle. **b** In situ hybridization showing αA-crystallin expression differences in the lens viewed from lateral and dorsal sides at 40 hpf. From [53]. **c** Differences in adult brain morphology viewed dorsally. *ot* optic tectum, *ol* olfactory lobe. **d** Differences in head morphology and anti-tyrosine hydroxylase staining showing differences in olfactory neuronal regions (on) in 6 dpf larvae. **e** Larval skeletons showing differences in rib number (r and box). **f** DASPEI stained larvae showing differences in cranial neuromast density. **g** Assay of VAB in the laboratory showing tracings of locomotory trajectories in chambers relative to the position of a vibrating rod (center). Also see Additional file 4: Movie S4. **h** Differences in jaw size and tooth number in adults. *te* teeth. **i** Isolated sclera stained for cartilage (blue) and bone (red) showing differences in size and ossicle formation (os). **j** Differences in feeding posture behavior with respect to the substrate (**f** and **g** reproduced from [7] with permission from Cell Press)

Most *Astyanax* research centers on the gain and loss of traits in cavefish compared to surface fish [10]. The gains include increases in the olfactory lobes, the hypothalamus, jaws, taste buds, teeth, neuromasts, body fat, and VAB, while the losses include eyes, melanin pigmentation, heart regenerative capacity, circadian rhythms, and social behaviors (Fig. 2). In addition, significant changes in metabolic processes, the shape and symmetry of craniofacial bones, the duration of sleep, and body posture during feeding occur between the two morphs (Fig. 2). We next comment on some of the major questions of broad significance that can be addressed in this model.

### Gain and loss of sensory modalities in the cave environment

**Fig. 3:**
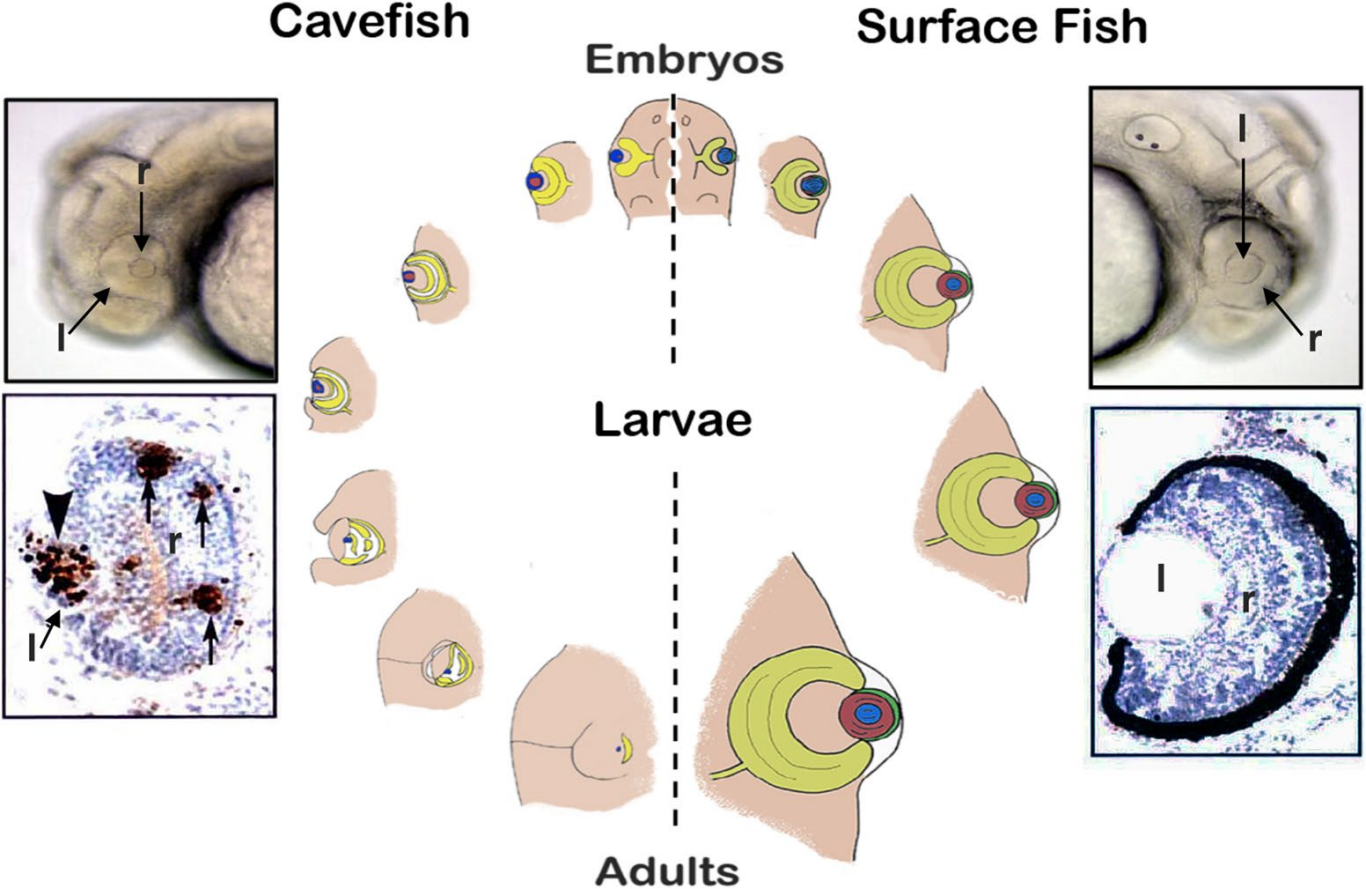
Eye development and degeneration. Right: surface fish eye develops with a normal lens (l) and retina (r) and no apoptosis. Left: cavefish eye begins to develop with a small lens and optic cup with a gap in the ventral portion of the retina, and later arrests in growth and disappears into the orbit. First the lens (arrowhead) and then parts of the retina (arrows) undergo apoptosis detected by TUNEL. Center: steps of eye development in surface fish (right) and eye degeneration in cavefish (left) (modified from [22])

The most famous cavefish trait is the loss of eyes, and how they are lost is an intriguing process [11]. Eyes begin to form in cavefish embryos, but suddenly stop growing and degenerate during later development (Fig. 3). Eye degeneration starts with apoptosis of the lens, which then spreads to the retina. New retinal cells continue to arise from stem cells in the ciliary marginal zone, but are subsequently removed by apoptosis before they differentiate [12], resulting in arrest of optic growth. Lens apoptosis is important in the overall control of eye degeneration (Fig. 3), as eye morphology can be restored by transplanting a surface fish lens into a cavefish optic cup [13]. The Shh and Fgf8 morphogens, which are overexpressed or appear precociously along the anterior midline during embryogenesis [14, 15], regulate eye loss. Due to Shh expansion in the prechordal region, *pax6* is suppressed in the overlying neural plate, and consequently smaller retinal fields are formed (Fig. 2a). Shh overexpression also affects eye development by inducing lens apoptosis through an unknown mechanism [16]. In parallel to eye degeneration, the gustatory and olfactory systems are expanded in cavefish. An antagonistic tradeoff exists between the loss of eyes and increased taste bud numbers and jaw size, which is controlled by Shh signaling centered in the developing oral area and taste bud primordia [16]. Accordingly, eyes can be reduced and taste buds and jaws increased in surface fish embryos by conditional overexpression of *shh*. Tradeoffs also link other trait gains and losses. The relationship between the olfactory and lens placodes is impacted by a tradeoff controlled by Shh, Fgf8, and BMP4 signaling [17], antagonism between eyes and number of teeth may be controlled by Fgf8, BMP4, and *pitx2* [18], the enlargement of the hypothalamus is mediated by re-deployment of cells from the ventral retina [15], and VAB (Fig. 2g) and increased cranial neuromast density may be facilitated (Fig. 2f) by the extra space created by eye loss [19]. The precise mechanisms responsible for sensory trait linkages are still poorly understood.

### Genetic architecture of trait evolution

**Fig. 4:**
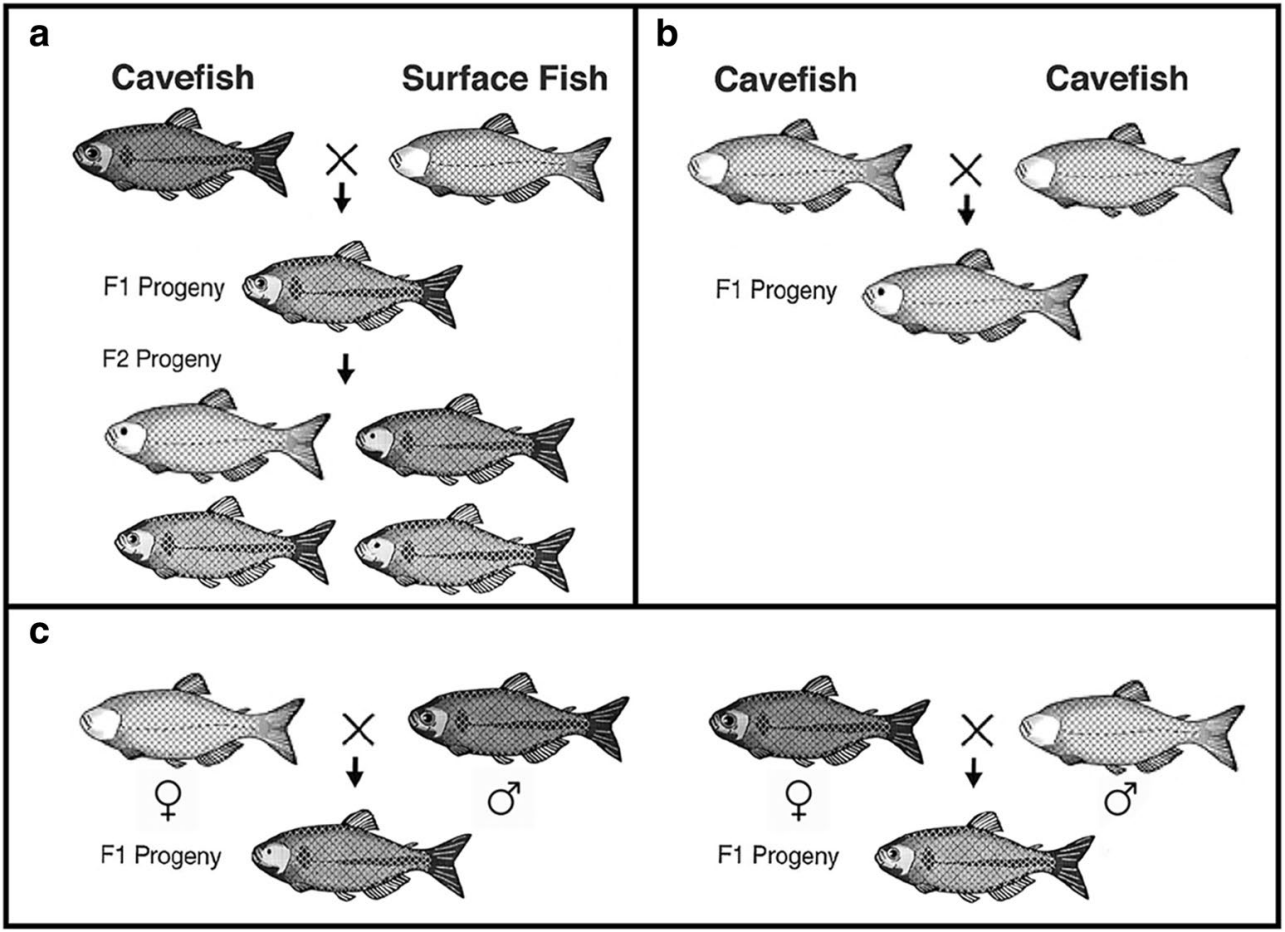
Crosses used in *Astyanax* genetics. **a** Cavefish × surface fish intercross showing F1 hybrid progeny with eyes and pigmentation (above) and subsequent F1 hybrid intercross showing F2 hybrid progeny with a broad range of eye sizes and 3:1 ratio of pigmented versus depigmented individuals (below). **b** Complementation cross between two different cavefish populations showing F1 hybrid progeny with partially restored eyes but no restoration of pigmentation. **c** Reciprocal (bidirectional female/male) cross showing F1 hybrid progeny with eye phenotypes suggesting maternal effects on eye degeneration

Because they are the same species, crosses between cavefish and surface fish are possible, and the hybrids can be used in QTL analysis to identify the genomic regions responsible for trait evolution (Fig. 4a). These studies have shown that eye loss is an additive trait controlled by more than a dozen QTL scattered across the genome [20, 21]. Thus far, *cbsa*, which affects eye growth by interfering with optic vasculature function, is the only mutated gene that has been positively identified under an eye QTL [22]. The loss of melanin pigmentation is caused by differentiation of fewer melanophores, reduced melanin synthesis, which leads to brown coloration in some cavefish populations (e.g., Tinaja), and the complete loss of melanin, which results in albinism in other cavefish populations (e.g., Pachón and Molino) [23]. Multiple QTL are also responsible for reduced pigmentation [20], but within this complex trait, two simple phenotypes, brown coloration and albinism, are controlled by single genes with classic Mendelian inheritance. QTL analysis has identified the *mc1r* gene as directly responsible for the brown phenotype [24] and the *oca2* gene for albinism [25]. The other genes underlying changes in pigmentation have not been identified. Genetic architectures for loss of sleep [26], schooling [27], feeding posture [28], and craniofacial asymmetry [29] are also polygenic, while the loss of scleral ossicles of the eyes is probably controlled by only two or three mutated genes [30]. Although QTL analysis provides the potential to identity the causal genes for trait changes, most of the existing QTL are large and contain hundreds of genes, making gene identification painstaking. The advent of CRISPR–Cas9 gene editing may provide more rapid assays for QTL genes and thus expedite progress.

### Use of the same or different genes during repeated evolution

The existence of cavefish populations evolving in parallel or by convergence from surface fish ancestors offers an excellent opportunity to study gene use during repeated evolution. Complementation crosses between different cavefish populations (Fig. 4b) have shown that genes controlling eye loss can be the same or unique in these populations [31]. The *cbsa* gene is mutated at the same *cis*-regulatory position in six different cavefish populations [22], suggesting that it functions repeatedly in eye loss. In some cavefish populations (e.g., Pachón, Tinaja) *cbsa* shows the same mutation, while in others (e.g., Molino) a different mutation occurs at the same site, revealing a possible DNA hotspot for mutational change. Likewise, the *mc1r* gene is mutated at the same position in the coding region of multiple cavefish populations [24], and the *oca2* gene shows large deletions at different places in the coding region in Pachón and Molino cavefish [25]. These studies show that the same genes harboring different mutations can be responsible for eye and pigment loss in different cavefish populations. Complementation studies are limited by the relatively low numbers of different cavefish populations that have been sampled in the wild and transferred to the laboratory.

### Role of maternal effects in trait evolution

The ability to carry out reciprocal hybridization (Fig. 4c)—the fertilization of cavefish eggs with surface fish sperm and vice versa—provides a novel way to distinguish between traits evolved under maternal or zygotic control [32]. While most trait changes have been considered to originate zygotically, degeneration of the lens and retina, temporal differences in gastrulation, and some changes in the brain are under direct maternal control, and thus have consequences extending into the larval period [32, 33]. The trait changes that occur via maternal effects could define early control points that broadly impact many downstream events in cavefish development.

### Specialized adaptations to nutrient-limited environments

The absence of primary productivity in dark caves and sporadic availability of nutrients from outside has led to the evolution of unique survival strategies in many cavefish populations. Feeding is excessive during the periods in which food is abundant. The increases in olfactory organs, taste buds, and cranial neuromast numbers help cavefish detect food in the dark. Feeding posture behavior also increases food finding in cavefish (Additional file 1: Movie S1 and Additional file 2: Movie S2). Using VAB (Additional file 3: Movie S3), cavefish detect and swim toward vibrations to consume living prey [7]. High appetites and continuous feeding allow weight gain and fat deposition during periods of food abundance for use during periods of famine [34]. Despite excessive fat accumulation, especially in the enlarged livers of some cavefish populations (e.g., Tinaja), there are no obvious differences in health or lifespan compared to surface fish. Low metabolic rates and resistance to weight loss help cavefish to survive during periods of low food availability [35]. The molecular mechanisms responsible for some of these physiological traits show unexpected similarities to human feeding disorders. Some cavefish populations harbor a nonsynonymous mutation in *mcr4r*, a target gene of the leptin pathway, and the same amino acid shift is associated with obesity in humans [35]. After excessive eating, cavefish experience diabetes-like high levels of blood sugar, but they are insulin resistant because of a mutation in the insulin receptor, which occurs at the same site as in human type 2 diabetics [36]. Cavefish thus offer unique possibilities to study the genetic basis of human metabolic diseases.

### Circadian rhythms in perpetual darkness

Cavefish are used as models to understand circadian activity in the timeless and dark cave environment [37]. In contrast to surface fish, cavefish do not show cyclic transcriptional activity in the clock gene *per1* in the wild [38]. But in the laboratory they can be can be entrained to undergo rhythmic *per1* expression by light cycles, although the periodicity differs from surface fish, suggesting that fundamental differences have evolved in core circadian processes. Circadian processes could be simply vestigial remnants of the ancestral surface fish clock or could be related to the use of clock-related genes in other fundamental cellular processes. A limitation is the difficulty in conducting circadian rhythm experiments in dark caves.

### Genetic basis of regeneration

*Astyanax* cavefish are being used to address the genetic basis of cardiac regeneration. The cavefish heart shows morphological and physiological differences from its surface fish counterpart [39]. Surface fish can efficiently repair induced cardiac injuries but cavefish hearts are unable to regenerate, leaving permanently scarred tissue [40]. RNA sequencing and QTL analysis have identified *lrrc10*, encoding a cardiac protein of unknown function, and several genes encoding extracellular matrix proteins, as candidates for controlling heart regeneration [40]. Additional comparisons of tissue and organ regeneration between the morphs could provide novel information in regenerative biology.

### Craniofacial and skeletal patterning

Cavefish have evolved dramatic changes in craniofacial and skeletal traits relative to surface fish, highlighted by differences in the number and shape of ocular bones and an unusual lateral bend of the skull [41]. Some cavefish populations also have fewer ribs than surface fish (Fig. 2f). Lens manipulations show that orbital bone shape is linked to reduced eye development [42]. Asymmetric patterning of the cranial neuromasts also impacts the formation of facial dermal bones. Different cavefish populations vary in the severity of craniofacial changes. Craniofacial asymmetry may be driven by requirements for navigation in darkness. Most craniofacial traits are genetically complex [29], and the identity of the underlying genes has not been resolved.

### Ecological integration

The relationship between traits expressed in the laboratory and field [43], interactions between cavefish and other cave fauna, including trophic interactions and parasitism, and the physical differences in cave ecosystems leading to adaptation are ripe for investigation. How surface fish first colonized the challenging cave environment, including the roles of standing and cryptic genetic diversity [44], phenotypic plasticity and genetic assimilation [45], and adaptive and neutral evolution [46], are also worthwhile pursuits. Limitations for ecological studies are related to the remoteness of the caves, the difficulties in studying cave habitats, and severe and dangerous cave flooding.

## Experimental approaches

### Comparative studies

Precise synchrony between developing morphs is advantageous for comparative studies [6]. Accordingly, the timing of differences and potential heterochrony [15] between surface fish, cavefish, and their hybrids can be investigated. This attribute also permits direct comparison of gene expression profiles.

### Hybridization and genetics

As proxies of wild type and natural “mutants”, the morphs provide a way to explore the genetics of trait development and evolution (Fig. 4). Hybridization can be accomplished via group mating, paired mating, or in vitro fertilization and is possible between surface fish and all cavefish populations or between the various cavefish populations. F2 progeny of surface fish × cavefish hybrids are used in QTL analysis (Fig. 4a) [20, 21, 24–30]. Crosses between different cavefish populations are used for determining whether traits can be restored and mutant genes complemented in F1 hybrids (Fig. 4b). Reciprocal hybridization (Fig. 4c) can be used to identify maternal effects [32]. Hybridization followed by artificial selection can generate strains with combined surface fish and cavefish traits [47], which are useful to investigate antagonistic tradeoffs [48].

### In situ hybridization, immunolocalization, and other assays

Small transparent *Astyanax* embryos enable effective whole mount in situ hybridization and antibody staining. In situ hybridization is done easily between the unfertilized egg and 4–5 dpf, but is more problematic as larvae increase in size and opaqueness. Undifferentiated melanophore lineage cells can be detected in albino fish by exogenous l-DOPA treatment [49].

### Tissue and organ manipulations

The lens, optic vesicles, and pineal organs can be ablated or transplanted [12, 13, 42, 50] (Additional file 4: Movie S4), eyes can be removed and stockpiled [51], and neural crest cells [52] can be exchanged between embryos and young larvae of the two morphs. It is also possible to swap lenses between surface fish and zebrafish (Additional file 4: Movie S4). Expansion of these methods could be used to address embryonic organizer activity, induction, and lineage allocations in an evolutionary context.

### Gene manipulations

Gene expression can be knocked down by injection of eggs with morpholinos and the combined use of judicious controls [16, 22, 53]. Gene knockouts at efficiencies of 50–75% are possible by microinjection of TALEN [54] or CRISPR–Cas9 [22, 55] into fertilized eggs, and mutant lines can be maintained by standard husbandry. Genetic complementation between CRISPR–Cas9 surface fish mutants and cavefish can be used to confirm the relationship of edited genes to the genes responsible for naturally evolved cavefish traits and to evaluate off-target effects [55]. Gene overexpression has been carried out by injection of synthetic mRNAs or a gene fusion construct containing the zebrafish temperature-inducible *hsp70* promoter into eggs [16, 22].

### Transgenesis

Transgenic mosaic *Astyanax* were created using the I-SceI-meganuclease system under control of a zebrafish β-b1-crystallin promoter or the Tol2 system under control of the *Xenopus* cardiac actin promotor with up to 77% efficiency [9]. Two stable transgenic lines have been developed using the zebrafish Tol2 system [56]. One line consists of the zebrafish ubiquitin promoter fused to GFP and the other is a Cntnap2-mCherry construct. The direct transfer of Tol2 constructs from zebrafish will expedite the generation of more transgenic lines.

## Research community and resources

### Reviews, book, and publications

Comprehensive reviews [11, 57] and a book [58] summarizing different topics in *Astyanax* biology and evolution have been published. Many other publications can be downloaded and bibliographies viewed in the AMCS website (www.Mexicancaves.org).

### Methods collection

A collection of methods is published in JOVE [59]. It includes protocols for raising and spawning fish, in vitro fertilization, in situ hybridization, antibody staining, lateral line staining with DASPEI, and methods for larval phenotype analysis.

### Brain atlas

An atlas consisting of serial sections of adult surface fish and Pachón, Tinaja, and Molino cavefish brains has been produced [60]. This atlas provides the opportunity to associate evolutionary changes in the brain with behavior.

### Genome resources

The annotated genome assemblies for *A. mexicanus* surface fish (“Mexican tetra” Astyanax_mexicanus-2.0, GCA_0003722685.2) and Pachón cavefish (“Pachon cavefish” AstMex102, Ensembl release 93) are on the Ensembl genome browser (www.useast.emsembl.org). These resources can be used to align QTL to genome sequences and search for candidate genes and indels [61].

### Meetings and conferences

AIM convenes every other year. AIM 2019 was attended by about 70 delegates. AIM programs with abstracts can be downloaded on the AMCS website. An official Astyanax community has been established [62], which has generated cooperation in genome sequencing [61] and other research projects. Some *Astyanax* researchers also attend the ISSB conferences (www.sibios-issb.org), which bring together researchers working on all cave organisms and caves.

### CAVEFIN website

A resource with information on AIM, AIM group photographs, news, job opportunities, lists of labs, principal investigators, publications, protocols, links to other cavefish research sites, and a discussion forum is online at https://research.stowers.org/cavefin/.

## Supplementary information

**Additional file 1: Movie S1.** Feeding of the surface morph in the laboratory. Sighted surface morphs feed in the water column. Credit: Mandy Ng.

**Additional file 2: Movie S2.** Feeding of the cave morph in the laboratory. Blind cave morphs feed by skimming the substrate using a unique feeding posture behavior. Credit: Mandy Ng.

**Additional file 3: Movie S3.** VAB studied in the laboratory. Pachón cavefish (left) and surface fish (right) swimming in assay chambers in the absence of a vibrating rod (top), the presence of a stationary rod (0 Hz, middle), and the presence of a 50 Hz vibrating rod (bottom). Credit: Masato Yoshizawa. Video reproduced from [7] with permission from Cell Press.

**Additional file 4: Movie S4.** Lens extirpation and transplantation. Credit: Yoshiyuki Yamamoto.

## Data Availability

Data sharing is not applicable to this article as no datasets were generated or analyzed during the current study.

## Abbreviations

AIM: Astyanax International Meeting
AMCS: Association for Mexican Cave Studies
BMP4: Bone morphogenetic protein 4
dpf: Days post-fertilization
DASPEI: (2-(4-(Dimethylamino)styryl)-*N*-ethylpyridinium iodide)
*cbsa*: *Cystathionine ß*-*synthase* gene
Cntnap2: Contractin-associated protein 2
CRISPR–Cas9: Clusters of regularly interspaced short palindromic repeats–CRISPR associated 9
Fgf8: Fibroblast growth factor 8
*hsp70*: *Heat shock protein 70* gene
GFP: Green fluorescent protein
hpf: Hours post-fertilization
ISSB: International Society for Subterranean Biology
JOVE: *Journal of Visualized Experiments*
l-DOPA: l-3,4-Dihydroxyphenylalanine
*lrrc10*: *Leucine rich repeat containing 10* gene
*mc1r*: *Melanocortin 1 receptor* gene
*mc4r*: *Melanocortin 4 receptor* gene
*pitx2*: *Paired*-*like homeodomain 2* gene
*pax6*: *Paired box 6* gene
*Sce1*: *Saccharomyces cerevisiae* endonuclease 1
*shh*: *Sonic hedgehog* gene
Shh: Sonic hedgehog protein
TALEN: Transcription activator-like effector nuclease
Tol2: Transposable element 2
TUNEL: Terminal deoxynucleotidyl transferase dUTP nick end labeling
VAB: Vibration attraction behavior
